# Ongoing liver inflammation in patients with chronic hepatitis C and sustained virological response

**DOI:** 10.1371/journal.pone.0171755

**Published:** 2017-02-14

**Authors:** Christoph Welsch, Mira Efinger, Michael von Wagner, Eva Herrmann, Stefan Zeuzem, Tania M. Welzel, Christian M. Lange

**Affiliations:** 1 Department of Internal Medicine 1, JW Goethe-University Hospital, Frankfurt/Main, Germany; 2 Institute of Biostatistics and Mathematical Modelling, Faculty of Medicine, Goethe-University, Frankfurt/Main, Germany; National Taiwan University Hospital, TAIWAN

## Abstract

**Background:**

Novel direct-acting antiviral DAA combination therapies tremendously improved sustained virologic response (SVR) rates in patients with chronic HCV infection. SVR is typically accompanied by normalization of liver enzymes, however, hepatic inflammation, i.e. persistently elevated aminotransferase levels may persist despite HCV eradication. Aim: To investigate prevalence and risk factors for ongoing hepatic inflammation after SVR in two large patient cohorts.

**Methods:**

This post-hoc analysis was based on prospectively collected demographic and clinical data from 834 patients with SVR after HCV treatment with either PegIFN- or DAA-based treatment regimens from the PRAMA trial (n = 341) or patients treated at our outpatient clinic (n = 493).

**Results:**

We observed an unexpected high prevalence of post-SVR inflammation, including patients who received novel IFN-free DAA-based therapies. Up to 10% of patients had ongoing elevation of aminotransferase levels and another 25% showed aminotransferase activity above the so-called healthy range. Several baseline factors were independently associated with post-SVR aminotransferase elevation. Among those, particularly male gender, advanced liver disease and markers for liver steatosis were strongly predictive for persistent ALT elevation. The use of IFN-based antiviral treatment was independently correlated with post-SVR inflammation, further supporting the overall benefit of IFN-free combination regimens.

**Conclusion:**

This is the first comprehensive study on a large patient cohort investigating the prevalence and risk factors for ongoing liver inflammation after eradication of HCV. Our data show a high proportion of patients with ongoing hepatic inflammation despite HCV eradication with potential implications for the management of approximately one third of all patients upon SVR.

## Introduction

Sustained virological response (SVR), defined as undetectable serum hepatitis C virus RNA (HCV-RNA) 12 to 24 weeks after the end of treatment, is the primary goal of antiviral therapy in patients with hepatitis C. With pegylated interferon-alfa (PEG-IFN-α) and ribavirin treatment, the former standard-of-care, SVR rates were approximately 50% in patients infected with HCV genotype 1 and 70–80% in those infected with genotype 2 or 3. The recent introduction of numerous directly acting antiviral agents (DAAs) against HCV enabled interferon-free, all-oral regimens which achieve SVR rates of at least 90% [1–2].

SVR is typically accompanied by normalization of aminotransferases (ALT, AST) as surrogate markers for hepatic inflammation, considered as patient relevant endpoint with a 60–80% reduction in the development of cirrhosis and hepatocellular carcinoma (HCC), respectively [3]. Aminotransferase elevations have been shown to be associated with an increased risk of mortality independent of etiology [4]. Poynard et al. observed 10 years after SVR that only about half of the patients with SVR and advanced baseline fibrosis had a significant improvement of liver fibrosis with some patients developing progressive liver disease with newly diagnosed liver cirrhosis and approximately 2.5% developing HCC. The net reduction of cirrhosis prevalence was only 5%. Despite viral cure, fibrosis seems to progress in more than 10% of patients [5]. A study by Innes et al. report that patients achieving an SVR were more than four times less likely to die from a liver-related reason than non-SVR patients, however, non-cirrhotic patients with an SVR reported to harbor a disproportionate burden of liver-related morbidity which was up to six times that of the general population [6].

Patient characteristics associated with risk of ongoing liver damage upon viral eradication are poorly understood and underlying disease mechanisms still remain to be characterized. In particular it is unknown whether the rate of elevated aminotransferases differs between patients having achieved an SVR with or without an IFN-based therapy.

Here we present a large, retrospective study on patients with persistently elevated aminotransferase levels upon achievement of SVR. We first identified the prevalence of post-SVR aminotransferase elevation (post-SVR inflammation) and explored patient and clinical characteristics potentially associated with ongoing liver disease.

## Patients and methods

### Patients

Cohort 1: 341 patients were enrolled into a discovery cohort to assess the frequency of ongoing liver inflammation in patients upon achievement of SVR. All patients were treated within the PRAMA trial, a randomized, multi-center, partially placebo-controlled Phase IV study comparing the efficacy and tolerability of a 48-week combined therapy with pegylated interferon-alfa-2a, ribavirin and amantadine sulphate versus placebo in previously untreated patients with CHC-genotype-1-infection (ClinicalTrials.gov identifier: NCT00127777). The study was performed between July 2003 and February 2007 at German centers. Patients included in this study were female and male subjects over 18 years of age and below 70 years of age with serological evidence of CHC infection, treatment naïve, and serum HCV-RNA quantification >600 IE/ml by means of quantitative HCV-RNA assay. Patients showed increased ALT serum level on at least one date within a 56-day screening phase prior to start of study medication. Patients with decompensated liver cirrhosis were not enrolled. ALT serum levels were determined at a follow-up visit 24 weeks post treatment.

Cohort 2: This cohort included 493 consecutive patients at our outpatient department at Goethe-University Hospital Frankfurt Germany with CHC who started antiviral treatment with either PEG-IFN-α + ribavirin therapy without direct-acting antiviral agent (DAA) or triple therapy, or treatment without interferon (all DAA combination therapies, all-oral), between January 2002 and May 2014. Patients included in this cohort had follow-up visits at 24 and 48 weeks after the end of treatment with a documented virological response (SVR) on either IFN-based or IFN-free treatment. Treated patients who were lost to follow-up before 24 weeks after end of treatment were, per definition, not able to attain SVR status before dropout. For this reason, these patients were not included in the analyses. The study is approved by the local ethics committee at Goethe University-Frankfurt.

### Study assessments

Data were obtained on patient demographics (gender, age), BMI (kg/m^2^), severity of fibrosis (if available; Ishak fibrosis score), previous treatment and recent antiviral therapy (type of medication, treatment period, virological response), as well as presence of diabetes mellitus, or reported alcohol consumption. The use of more than 30 g/d (men) or 20 g/d (women) of alcohol was considered severe alcohol consumption, and those subjects were excluded from study participation. Other markers assessed at baseline were: anti-nuclear antibodies [ANA], anti-mitochondrial antibodies [AMA], anti-smooth-muscle antibodies [ASMA], liver-kidney microsome antibodies [LKM], soluble-liver antigene antibodies [SLA], liver-pancreas antibodies [LP], and immunoglobulines (IgG) for autoimmune diseases. Furthermore, we assessed virological data, HCV genotype, HCV-RNA levels, anti-hepatitis B core antigen and HIV antibodies, as well as bilirubin, albumin, creatinine, platelets, international normalized ratio for prothrombin time [INR], aspartate aminotransferase [AST], alanine aminotransferase [ALT] and gamma-glutamyl transferase [GGT] levels at baseline.

Bilirubin, creatinine, ALT levels, GGT, autoantibodies and HCV-RNA levels were also assessed at week 24 and 48 follow-up visits (after end of treatment).

### Clinical outcome measures

The primary outcome measure of the study was ongoing elevation of the aminotransferase ALT upon achievement of SVR (post-SVR inflammation) at follow-up week 24 and 48.

### Statistical analyses

Univariate and multivariate analyses examined associations between clinical and laboratory factors in patients with SVR and ongoing aminotransferase elevation. Baseline characteristics were compared between patients who had received IFN-based versus IFN-free treatment regimens using the χ^2^ contingency tables or Wilcoxon-Mann-Whitney U tests, as appropriate. Associations between serum aminotransferases as continuous or categorical variables were assessed in linear and logistic regression models, respectively. After univariate analyses, multivariate analyses were performed for significant associations. Multivariate analyses were obtained by using backward selection, using a P value ≥0.15 for removal from the model. Only patients with complete data for the remaining covariates were included in multivariate analyses. All statistical tests were 2-sided and P values <0.05 were considered to be significant.

### Ethics approval

The study is approved by the local ethics committee at Goethe University-Frankfurt. The study is approved by the Goethe University-Frankfurt ethics committee. For this retrospective analysis, all data were anonymized and deidentified. No informed consent was required for this retrospective analysis.

## Results

For the present study, ALT levels were assessed at baseline (before antiviral treatment), as well as 24 weeks post treatment in a discovery cohort (cohort 1) and a replication cohort (cohort 2), respectively, as well as 48 weeks post treatment in the replication cohort 2. Elevated ALT levels are defined as derived from the updated definitions by Prati et al. [7–8]. According to that, we differentiate between healthy ALT levels, that is <20 U/mL for women and <31 U/mL for men, and normal ALT levels that are below the upper limit of normal (ULN) but above the healthy range. For elevated ALT levels, we applied a threshold of <50 U/mL as ULN.

### Cohort 1 –Baseline characteristics and post-SVR aminotransferase activity

Cohort 1 consisted of 341 patients with compensated chronic HCV genotype 1-infection who achieved an SVR after 48 weeks of combination therapy with PEG-IFN-α 2a, ribavirin and amantadine sulphate or placebo (Table 1). Of the 341 patients in this cohort 48% were male. The mean age was 44 years.

**Table 1:** Baseline characteristics of included patients.

Characteristics	Cohort 1	Cohort 2		P value^b^
	(N = 341)	IFN-based^a^ (N = 318)	IFN-free (N = 175)	
Male gender, n (%)	164 (48)	194 (61)	90 (51)	n.s.
Age (years), mean (IQR)	44 (35–53)	48 (39–55)	53 (45–61)	<0.0001
Diabetes, n (%)	12 (4)	28 (9)	17 (10)	n.s.
Sodium (mmol/L), mean (IQR)	139 (138–141)	141 (140–143)	141 (139–142)	0.08
Creatinine (mg/dL), mean (IQR)	0.9 (0.8–1.0)	0.86 (0.72–0.92)	0.85 (0.70–0.95)	n.s.
Bilirubin (mg/dL), mean (IQR)	0.6 (0.4–0.7)	0.72 (0.5–0.8)	0.8 (0.4–0.9)	n.s.
ALT (U/L), mean (IQR)	77 (49–92)	97 (45–119)	77 (41–95)	0.006
AST (U/L), mean (IQR)	121 (65–152)	68 (37–84)	66 (36–76)	n.s.
γGT (U/l), mean (IQR)	-	91 (28–107)	96 (30–110)	0.5
HCV RNA (Mio IU/mL), mean (IQR)	1.8 (0.6–5.5)	3.7 (1.0–7.3)	3.9 (1.2–6.9)	0.02
BMI, mean (IQR)	25 (22–27)	24 (22–28)	25 (22–28)	0.8
Albumin (g/dL), mean (IQR)	4.2 (4.0–4.4)	4.5 (4.3–4.7)	4.2 (4.0–4.6)	<0.0001
INR, mean (IQR)	-	1.04 (0.97–1.08)	1.06 (0.94–1.10)	n.s.
Platelets (per nl), mean (IQR)	231 (185–267)	201 (157–245)	200 (158–247)	n.s.
HCV Genotype				
1/4, n (%)	341 (100)	210 (67)	146 (84)	<0.001
2/3, n (%)		104 (33)	27 (16)	
Cirrhosis, n (%)	6 (2)	37 (12)	48 (28)	<0.001

ALT, alanine aminotransferase; AST, aspartate aminotransferase; BMI, body mass index; γGT, γ-glutamyl transferase; INR, international normalized ratio; IQR, interquartile range; n.s., not significant.

^a^ 182 and 145 patients received PEG-IFN-α + ribavirin therapy without DAAs and triple therapy (PEG-IFN-α + ribavirin + DAA), respectively.

^b^ Baseline characteristics of the replication cohort were compared between patients who had received IFN-based versus IFN-free treatment regimens.

Elevated ALT levels during follow-up were observed in 23 patients (6.7%) of the cohort 1. Healthy ALT levels, however, were only observed in 155 patients (45.5%), whereas the majority of patients, 163 (47.8%), showed normal ALT levels (S1 Table). Hence a significant proportion of SVR patients from this cohort showed ongoing elevation of ALT levels despite viral eradication while the majority of patients showed normal ALT levels that potentially requires further surveillance.

### Cohort 2– Baseline characteristics

Cohort 2 of our study consisted of 493 patients with SVR. Patients in this cohort were treated IFN-based, either with PEG-IFN-α + ribavirin therapy without DAAs (173 patients, 35%) or triple therapy, that is, PEG-IFN-α + ribavirin + DAA (145 patients, 30%), or were treated IFN-free using all-DAA regimens (175 patients, 35.5%).

The demographic and baseline clinical characteristics in cohort 2 of the 318 patients that were treated IFN-based and the 175 patients that were treated IFN-free were generally balanced (Table 1). Sixty-one percent of the patients with IFN-based therapy were male gender versus 51% with IFN-free therapy. The mean age was 48 years versus 53 years, respectively.

The majority of patients in the IFN-free population was infected with HCV genotypes 1 or 4 (84%) versus genotype 2 or 3 (16%), a discrepancy that was less prominent in the IFN-based population (67% versus 33%, respectively). Patients with cirrhosis were more frequent in the IFN-free population (28%) than in the IFN-based population (12%).

In comparison to the cohort 1, the cohort 2 (IFN-based and IFN-free) showed higher incidence for diabetes, 4% versus 9% and 10%, respectively, and liver cirrhosis, 2% versus 12% and 28%, respectively (Table 1).

### Cohort 2 and post-SVR aminotransferase activity

The overall frequency of elevated ALT levels in cohort 2 (IFN-based and IFN-free) was assessed at 24 weeks and 48 weeks post treatment (Table 2). Of the 493 patients in the cohort 2, data from 441 patients (89.4%) were available at week 24 and data from 319 patients (64.7%) were available at week 48.

**Table 2:** Frequency of elevated ALT levels after HCV eradication in cohort 2.

	ALT (U/mL) <20 (women); <31 (men)^a)^	ALT (U/mL) ≥20—<50 (women); ≥31—< 50 (men)^a)^^,^ ^b)^	ALT (U/mL) ≥50^b)^
**24 weeks post treatment N (%)**	279 (64.9)	118 (27.4)	33 (7.7)
**48 weeks post treatment N (%)**	206 (65.4)	78 (24.8)	31 (9.8)

ALT, alanine aminotransferase.

^a)^ The threshold of <20 and <31 for women and men, respectively, were derived from the updated definitions by Prati et al. [8]

^b)^ The threshold of <50 was defined as the upper limit of normal.

Of the 441 patients at week 24 post treatment, elevated ALT levels were observed in 33 patients (7.7%). Healthy ALT levels were observed in 279 patients (64.9%) with 118 patients (27.4%) showing normal ALT levels (Table 2). At week 48, healthy ALT levels were observed in 206 patients (65.4%), normal ALT levels in 78 patients (24.8%) and elevated ALT levels in 31 patients (9.8). Overall, the prevalence of elevated ALT levels in the replication cohort was close to 10% despite SVR with another approx. 25% of patients with SVR showing normal ALT levels. The rates of patients with healthy, normal and elevated ALT levels seem not to change significantly during 48 weeks of follow-up after achievement of SVR. A subgroup analysis in patients treated either with or without IFN showed higher prevalence of elevated ALT levels in patients that were treated IFN-based. At week 24 post treatment 29 patients (10%) treated IFN-based versus only 4 patients (3%) treated IFN-free showed elevated ALT levels as well as 29 patients (12%) versus 2 patients (3%) at week 48 respectively (S1 and S2 Tables).

### Predictors of post-SVR aminotransferase elevation

Univariate analysis (P, beta [SD beta]) showed that male gender (0.002, 6.67 [2.12]), low platelets (0.01, -0.04 [0.01]), BMI (0.007, 0.43 [0.16]) and IFN-based versus IFN-free therapy (0.04, -4.24 [2.01]) were the strongest predictors of elevated ALT levels, assessed as continuous variable at week 24 post treatment (Table 3). Additional variables which were significantly associated with post-SVR elevated ALT were baseline ALT (0.04, 0.03 [0.01]) and GGT (0.03, 0.01 [0.006]) levels. A subsequent multivariate analysis showed that these factors were all independent predictors of elevated ALT serum levels at week 24 post treatment (Table 4). Comparable results were obtained for linear and logistic regression analyses of ALT levels at 24 and 48 weeks post treatment (Tables 3 and 4). Logistic regression subgroup analysis in patients treated with or without IFN revealed baseline ALT, platelets and BMI as predictors of elevated ALT levels at week 24 post treatment in patients treated with IFN. In patients treated without IFN only platelets is identified as independent baseline predictor of elevated ALT at week 24 post treatment (S3 Table). We did not observe a correlation of a specific DAA or all-DAA combination therapy with post-SVR elevated ALT levels (data not shown). No correlation for liver-specific auto-antibodies and serum IgG with post-SVR elevated ALT levels was observed (data not shown).

**Table 3:** Linear regression analyses of alanine aminotransferase after HCV eradication.

	Univariate analysis		Multivariate analysis	
	beta (SD beta)	P	beta (SD beta)	P
**ALT (linear) at week 24 post treatment**				
Age (years, continuous)	-0.16 (0.08)	0.8		
Male gender	6.67 (2.12)	0.002	-6.75 (2.11)	0.002
Diabetes (presence)	1.38 (3.21)	0.7		
Bilirubin (mg/dL, continuous)	0.13 (1.77)	0.9		
ALT (U/L, continuous)	0.03 (0.01)	0.04	0.03 (0.01)	0.03
γGT (U/L, continuous)	0.01 (0.006)	0.03	0.01 (0.006)	0.02
Platelets (/nl, continuous)	-0.04 (0.01)	0.01	-0.04 (0.014)	0.008
HCV genotype 2, 3 versus 1, 4	-0.70 (2.29)	0.8		
IFN-free versus IFN-based therapy	-4.24 (2.01)	0.04	-4.23 (1.97)	0.03
BMI (kg/m^2^, continuous)	0.43 (0.16)	0.007	0.43 (0.16)	0.006
**ALT (linear) at week 48 post treatment**				
Age (years, continuous)	-0.08 (0.09)	0.3		
Male gender	5.90 (2.60)	0.02	-7.11 (2.49)	0.005
Diabetes (presence)	2.54 (4.30)	0.5		
Bilirubin (mg/dL, continuous)	-2.94 (2.41)	0.2		
ALT (U/L, continuous)	0.02 (0.02)	0.3		
γGT (U/L, continuous)	0.03 (0.01)	0.5	0.04 (0.01)	0.001
Platelets (/nl, continuous)	-0.04 (0.02)	0.04	-0.04 (0.02)	0.03
HCV genotype 2, 3 versus 1, 4	-4.44 (2.40)	0.06		
IFN-free versus IFN-based therapy	-3.02 (2.64)	0.2		
BMI (kg/m^2^, continuous)	0.19 (0.19)	0.06		

ALT serum concentration was analyzed as continuous variable in this model. ALT, alanine aminotransferase; BMI, body mass index; γGT, γ-glutamyl transferase; IFN, interferon; INR, international normalized ratio; IFN, interferon; ULN, upper limit of normal.

**Table 4:** Logistic regression analyses of alanine aminotransferase after HCV eradication.

	Univariate analysis		Multivariate analysis	
	OR (95% CI)	P	OR (95% CI)	P
**ALT (<20 (w) / <31 (m)) at week 24 post treatment**				
Age (years, continuous)	0.99 (0.97–1.02)	0.6		
Male gender	1.02 (0.56–1.84)	0.9	2.52 (1.17–5.44)	0.02
Diabetes (presence)	0.49 (0.18–1.29)	0.2		
Bilirubin (mg/dL, continuous)	0.89 (0.49–1.60)	0.7		
ALT (U/L, continuous)	1.01 (1.00–1.01)	0.002		
γGT (U/L, continuous)	1.01 (1.00–1.01)	0.002	1.01 (1.00–1.01)	0.006
Platelets (/nl, continuous)	0.99 (0.98–0.99)	0.0003	0.99 (0.98–0.99)	0.002
HCV genotype 2, 3 versus 1, 4	1.09 (0.57–2.08)	0.7		
IFN-free versus IFN-based therapy	0.51 (0.27–0.99)	0.048	0.51 (0.24–1.09)	0.08
BMI (kg/m^2^, continuous)	1.04 (0.99–1.01)	0.15	1.06 (0.99–1.12)	0.07
**ALT (≥50) at week 48 post treatment**				
Age (years, continuous)	0.98 (0.93–1.02)	0.3		
Male gender	0.20 (0.04–0.91)	0.03		
Diabetes (presence)	0.64 (0.13–3.10)	0.5		
Bilirubin (mg/dL, continuous)	0.67 (0.18–2.44)	0.5		
ALT (U/L, continuous)	1.00 (0.99–1.01)	0.05		
γGT (U/L, continuous)	1.00 (0.99–1.01)	0.08		
Platelets (/nl, continuous)	0.99 (0.98–0.99)	0.02	0.99 (0.98–1.00)	0.06
HCV genotype 2, 3 versus 1, 4	1.68 (0.58–4.88)	0.3		
IFN-free versus IFN-based therapy	0.13 (0.02–0.99)	0.04	0.15 (0.02–1.19)	0.07
BMI (kg/m^2^, continuous)	0.99 (0.91–1.07)	0.8		

ALT serum concentration was analyzed as categorical variable in this model, as indicated. w, women; m, men; ALT, alanine aminotransferase; BMI, body mass index; γGT, γ-glutamyl transferase; IFN, interferon; INR, international normalized ratio; IFN, interferon; ULN, upper limit of normal.

## Discussion

Sustained virological response is the major goal in antiviral treatment for chronic hepatitis C which to date is achieved for most patients with novel treatment regimens. Typically accompanied by normalization of aminotransferase levels, SVR is considered a patient relevant endpoint. Some patients in particular those with advanced liver disease and cirrhosis, however, show persistent liver injury even years after HCV cure [9]. Despite obvious clinical importance, the prevalence of elevated aminotransferase levels upon achievement of SVR and characteristics of respective patient populations are unknown. Here we present data from an observational study on post-SVR aminotransferase activity from two large cohorts with overall 834 patients after antiviral treatment with or without interferon (IFN).

The major finding from our study is the high prevalence of post-SVR elevated ALT levels despite viral eradication, including patients that have been treated with novel IFN-free direct-acting antiviral-based therapy. This observation supports our notion that ongoing aminotransferase elevation upon SVR is not a rare clinical event. Up to 10% of our patients were observed with persistently elevated ALT levels. Importantly, only approx. 65% of SVR patients showed healthy ALT levels upon viral cure, whereas another approx. 25% of SVR patients showed normal ALT levels that are below the upper limit of normal but above the so-called healthy range and hence require further surveillance [8]. Although baseline ALT activity showed significant correlation with ongoing ALT elevation, the correlation was only weak, and hence is not considered a reliable predictor for post-SVR aminotransferase activity.

In line with previous observations on the natural course of CHC infection, we found that male gender significantly correlated with post-SVR elevated ALT. A similar finding was previously reported in a study of chronic HCV patients from the Observatoire de i'Hépatite C-(OBSVIRC) population, the Cohorte Hépatite C Pitié-Salpêtrière (DOSVIRC) population, and the original METAVIR population. In this study several host factors were identified with strong association to fibrosis progression prior to IFN treatment, in particular male gender [10]. The authors, however, were unable to explain this association, but found male gender associated with a younger age at infection, shorter duration of infection, and daily alcohol consumption of more than 50 g, as well as a history of intravenous drug use. Such confounders on the course of disease are difficult to assess but potentially also relate to the gender-specific prevalence of persistently elevated ALT levels upon virus eradication that we report in our study albeit patients with significant alcohol consumption were excluded from our analysis.

Post-SVR inflammation in our study was observed in patients irrespective of being treated with IFN-based of IFN-free regimens. According to our data, IFN-based treatment per se can be considered as independent risk factor for persistently elevated ALT levels after SVR. We observed a strong correlation of IFN-based therapy with elevated ALT levels at week 24 and week 48 post SVR, further supporting the clinical benefits of novel IFN-free treatment regimens. Hence our data provide novel details on long-term adverse effects of IFN. Importantly, we also observed post-SVR inflammation in patients upon IFN-free all-DAA combination treatment. However no correlation between post-SVR ALT activity and any specific DAA or DAA combination is found, although NS3 protease inhibitors are reported previously to be associated with hepatotoxicity and elevated aminotransferase levels [11].

Moreover, we found body mass index and GGT significantly correlated with ongoing ALT elevations in our study. Serum ALT activity was already previously reported as independently related to body mass index [7]. The body mass index is associated with hepatic steatosis in CHC and known to affect the natural course of HCV infection [12], i.e. fibrosis progression [13–14], and is also a possible mediator of increased risk to develop type 2 diabetes [15]. A recent study reported a significant weight gain in 44% of patients with SVR12 upon IFN-free antiviral therapy [16]. Weight-gain after SVR potentially leads to deterioration of liver steatosis and might underlie elevated aminotransferase levels upon viral eradication. Further prospective investigations are needed to investigate a potential association between weight-gain after SVR and development of NASH or worsening of pre-existing NASH in patients with high normal or elevated BMI at baseline. Given the high prevalence of NASH in the western world, this would have important clinical implications for the post-SVR management of those HCV patients with liver steatosis.

Baseline platelet counts that are considered as predictors of a fibrotic stage in liver disease [17] were observed with a significant inverse correlation with post-SVR elevated ALT levels both in uni- and multivariate analyses. Hence, laboratory markers for fatty liver disease and/or advanced liver disease are independently related with serum ALT activity. A recent paper by York et al. reported that type I IFN-signaling impairs the balance in metabolism of cholesterol and long chain fatty acids [18]. The study reveals an important metabolic-inflammatory circuit that links cholesterol biosynthesis with activation of innate immunity, which might relate to our clinical observations with elevated aminotransferase levels in the long-term follow up of patients upon SVR. Our data can be interpreted as another argument in support of treating CHC patients early in order to avoid advanced stages of liver disease, not only to reduce the higher risk of treatment failure in cirrhotic patients but also to avoid potential disease progression upon SVR [19].

Strengths of the study are the large patient number and good data quality. Recall bias (e.g. for self-reported variables such as alcohol consumption) is unlikely as data were prospectively collected. Our study, however, has potential limitations. Follow-up data to explore the long-term impact of ALT elevations on morbidity (i.e. fibrosis progression, HCC risk) and mortality of patients enrolled into the PRAMA trial were not available. The follow-up of DAA-treated patients is currently ongoing but yet too short to evaluate clinical long-term outcomes, however, some studies suggested an increased risk of mortality in patients with elevated liver enzymes independent of etiology [4]. As liver biopsy is not standard of care for the management of most patients with chronic hepatitis C virus infection as well as due to the retrospective nature of our study, a comparison of pre-/post-treatment histologies was not possible within this large patient cohort. Also, body-weight was not routinely collected during follow-up in most patients.

Our study is the first clinical characterization of post-SVR aminotransferase elevation with an unexpected high prevalence that affect approximately one third of all patients upon viral eradication. Since CHC is both a virologic and fibrotic disease [20–21], the high prevalence of ongoing liver inflammation likely impact the future course of disease, cirrhosis progression and cancer development in thousands of patients with so far unrecognized burden to public health worldwide. Baseline factors that were (i) significantly associated and (ii) strongly correlated with ongoing elevation of ALT levels after SVR were male gender, the use of IFN in antiviral treatment regimens, and markers of advanced liver disease or steatosis. We observed body weight as significant baseline predictor that justify clinical concerns as high body weight might favor the development or worsening of pre-existing fatty liver disease or steatohepatitis (NASH) upon successful antiviral treatment. Due to the enormous implications for patient management after SVR, prospective studies are currently ongoing to further investigate the possible association between SVR and worsening of metabolic liver disease, as indicated herein.

In summary, this is the first comprehensive study on a large patient cohort investigating the prevalence and risk factors for ongoing liver inflammation after eradication of HCV. Our data show a high proportion of patients with ongoing hepatic inflammation despite HCV eradication with potential implications for the management of approximately one third of all patients upon SVR.

## Supporting information

S1 Table Frequency of elevated ALT levels after HCV eradication in the replication cohort: Subgroup analysis of patients treated with IFN-based antiviral therapy. (DOCX)

S2 Table Frequency of elevated ALT levels after HCV eradication in the replication cohort: Subgroup analysis of patients treated with IFN-free antiviral therapy. (DOCX)

S3 Table Logistic regression analyses of alanine aminotransferase after HCV eradication: Subgroup analysis of patients treated with IFN-based or IFN-free antiviral therapy. (DOCX)

## Abbreviations

ALT: alanine aminotransferase
AMA: anti-mitochondrial antibodies
ANA: anti-nuclear antibodies
ASMA: anti-smooth-muscle antibodies
AST: aspartate aminotransferase
BMI: body mass index
CHC: chronic hepatitis C
DAA: direct-acting antiviral agent
GGT: gamma-glutamyl transferase
HCC: hepatocellular carcinoma
HCV: hepatitis C virus
IFN: interferon
INR: international normalized ratio
LKM: liver-kidney microsome antibodies
LP: liver-pancreas antibodies
NASH: non-alcoholic steatohepatitis
RNA: ribonucleic acid
SLA: soluble-liver antigene antibodies
SVR: sustained-virological response
ULN: upper limit of normal
