# Supplementary material for: Ongoing liver inflammation in patients with chronic hepatitis C and sustained virological response
Source: PLoS One. 2017 Feb 14;12(2):e0171755. doi: 10.1371/journal.pone.0171755 (PMC5308806; doi:10.1371/journal.pone.0171755)
Supplement: S1 Table — (DOCX) [file pone.0171755.s001.docx]

**S1Table. Frequency of elevated ALT levels after HCV eradication in the replication cohort: Subgroup analysis of patients treated with IFN-based antiviral therapy.**

|  | **ALT (U/mL)<20 (women);<31 (men)^a)^** | **ALT (U/mL)≥20 - <50 (women); ≥31 - < 50 (men)^a), b)^** | **ALT (U/mL)≥50^b)^** |
| --- | --- | --- | --- |
|  |  |  |  |
| **24 weeks post treatment N (%)** | 181 (63) | 79 (27) | 29 (10) |
|  |  |  |  |
|  |  |  |  |
| **48 weeks post treatment N (%)** | 153 (64) | 58 (24) | 29 (12) |
|  |  |  |  |

ALT, alanine aminotransferase. a) The threshold of <20 and <31 for women and men, respectively, were derived from the updated definitions by Prati et al. [8] b) The threshold of <50 represents the upper limit of normal according to our laboratory.
