# Supplementary material for: Ongoing liver inflammation in patients with chronic hepatitis C and sustained virological response
Source: PLoS One. 2017 Feb 14;12(2):e0171755. doi: 10.1371/journal.pone.0171755 (PMC5308806; doi:10.1371/journal.pone.0171755)
Supplement: S3 Table — (DOCX) [file pone.0171755.s003.docx]

**S3 Table. Logistic regression analyses of alanine aminotransferase after HCV eradication: subgroup analysis of patients treated with IFN-based or IFN-free antiviral therapy.**

|  |  | **Univariate analysis** | |  | **Multivariate analysis** | | |
| --- | --- | --- | --- | --- | --- | --- | --- |
|  |  | **OR (95% CI)** | ***P*** |  | **OR (95% CI)** | ***P*** | |
| ***ALT (≥50) week 24 post treatment with IFN-based regimens*** | | | | | | | |
| Age (years, continuous) |  | 1.01 (0.98-1.04) | 0.3 |  |  |  | |
| Male gender |  | 0.78 (0.41-1.50) | 0.5 |  |  |  | |
| Diabetes (presence) |  | 0.88 (0.33-2.38) | 0.8 |  |  |  | |
| Bilirubin (mg/dL, continuous) |  | 2.03 (0.93-4.42) | 0.07 |  |  |  | |
| ALT (U/L, continuous) |  | 1.01 (1.01-1.01) | 0.009 |  | 1.00 (0.99-1.01) | 0.07 | |
| γGT (U/L, continuous) |  | 1.00 (0.99-1.00) | 0.09 |  |  |  | |
| Platelets (/nl, continuous) |  | 0.99 (0.98-0.99) | 0.005 |  | 0.99 (0.98-0.99) | 0.01 | |
| HCV genotype 2, 3 *versus* 1, 4 |  | 0.62 (0.35-1.21) | 0.2 |  |  |  | |
| BMI (kg/m^2^, continuous) |  | 1.08 (1.02-1.16) | 0.01 |  | 1.10 (1.02-1.18) | 0.006 | |
|  |  |  |  |  |  |  | |
| ***ALT (≥50) week 24 post treatment with IFN-free regimens*** | | | | | | | |
| Age (years, continuous) |  | 1.00 (0.97-1.03) | 0.9 |  |  |  | |
| Male gender |  | 1.42 (0.65-3.10) | 0.4 |  |  |  | |
| Diabetes (presence) |  | 0.44 (0.13-1.43) | 0.2 |  |  |  | |
| Bilirubin (mg/dL, continuous) |  | 1.43 (0.89-2.31) | 0.1 |  |  |  | |
| ALT (U/L, continuous) |  | 0.99 (0.99-1.00) | 0.7 |  |  |  | |
| γGT (U/L, continuous) |  | 1.00 (0.99-1.00) | 0.4 |  |  |  |  |
| Platelets (/nl, continuous) |  | 0.99 (0.99-1.00) | 0.1 |  | 0.99 (0.99-1.00) | 0.1 | |
| HCV genotype 2, 3 *versus* 1, 4 |  | 0.95 (0.32-2.82) | 0.9 |  |  |  | |
| BMI (kg/m^2^, continuous) |  | 1.02 (0.94-1.10) | 0.6 |  |  |  | |

ALT serum concentration was analyzed as categorical variable in this model, as indicated. w, women; m, men; ALT, alanine aminotransferase; BMI, body mass index; γGT, γ-glutamyl transferase; IFN, interferon; INR, international normalized ratio; IFN, interferon; ULN, upper limit of normal.
